# Unsupervised Functional Capacity Testing at Home: A Systematic Review

**DOI:** 10.1186/s40798-026-01029-6

**Published:** 2026-05-27

**Authors:** Debbie de Graaf, Alex J. van ’t Hul, Stella M. M. Thissen, Nienke M. de Vries, Bastiaan R. Bloem

**Affiliations:** 1 Donders Institute for Brain, Cognition and Behavior, Department of Neurology, Center of Expertise for Parkinson and Movement Disorders, R914, Radboud University Medical Center, Postbus 9101, 6500 HB Nijmegen, The Netherlands; 2 Radboud Institute for Health Sciences, Department of Respiratory Diseases, Radboud University Medical Center, Nijmegen, The Netherlands; 3 Department of Primary and Community Care, Radboud University Medical Center, Nijmegen, The Netherlands

**Keywords:** Functional capacity, Chronic disease, Remote, Home-based, Telemedicine

## Abstract

**Background:**

Physical functioning is closely linked to disability and mortality in older people. An important aspect of physical functioning is functional capacity, which can be measured with wearable technology. It is, however, unclear which tests can be completed using wearable technology and what the clinimetric properties of such measurements are. The objective was to systematically review home-based functional capacity tests and their clinimetric properties in people with a chronic disease.

**Methods:**

PubMed and CINAHL were used to find relevant articles. Clinical trials studying clinimetric properties including people with any type of cancer, chronic cardiovascular, pulmonary, or neurological disorder or people undergoing elective surgery were included in this review. Additionally, studies using an unsupervised or remotely supervised home-based functional capacity test were included. Information on clinimetric properties was extracted by two independent raters using the COSMIN guidelines. Relevant information was registered.

**Results:**

We included 29 articles, studying the 6-Minute Walk Test (6MWT) (*n* = 19), the Timed-Up-and-Go (*n* = 6), the 30 s Sit-To-Stand test (*n* = 2) and the step test (*n* = 2). The 6MWT with a smartphone-based application showed acceptable results for validity and reliability. For most other approaches, there was limited information available on clinimetric properties.

**Conclusions:**

This review shows that at home testing of functional capacity is feasible, although evidence on its clinimetric properties remains limited. The 6MWT has been studied most widely, showing acceptable results when performing the test using smartphone applications in people with chronic diseases. Further research is needed before these tests can be widely applied in both research and clinical care.

**Supplementary Information:**

The online version contains supplementary material available at 10.1186/s40798-026-01029-6.

## Key Points

- Knowledge about the clinimetric properties of functional capacity tests at home using different approaches is limited.
- The 6MWT has been studied most widely, showing acceptable results when performing the test using smartphone applications in people with chronic diseases. The results for the measurement properties of other approaches were inconclusive.

**Supplementary Information:**

The online version contains supplementary material available at 10.1186/s40798-026-01029-6.

## Background

Physical functioning is defined as the ability to carry out activities, including simple everyday tasks as well as more complex actions in various settings [1–5]. Physical functioning is an independent predictor of functional independence, disability, morbidity and mortality in healthy older people and in people with chronic diseases [4–11]. An important aspect of physical functioning is functional capacity, which can be defined as what a person can do in a standardized test environment [6]. Functional capacity is essential for maintaining a healthy and active life and has an impact on the physical, mental and social well-being of individuals [3, 6]. Therefore, monitoring functional capacity is important, especially in people with a chronic disease.

The gold standard to assess functional capacity is the measurement of peak oxygen uptake during a maximal cardiopulmonary exercise test [7]. This test is performed in clinical settings and requires advanced equipment and trained personnel, which makes it expensive and difficult to apply on a large scale. Therefore, submaximal field exercise tests have been developed for in-clinic use that do not require advanced equipment, including the 6-Minute Walk Test (6MWT), Timed Up and Go (TUG) test, Sit to Stand (STS) test and step test [12–18].

In these tests, participants are asked to perform a standardized test, such as walking as far as possible in six minutes (6MWT), standing up from a chair, walking a short distance, turning and sitting down again (TUG), repeatedly standing up and sitting down (STS), or stepping up and down on a step for a given time (step test). Performing these tests requires minimal equipment: a stopwatch, a chair or step, and a suitable space or straight walking course. Close observations and manual recordings by a clinician are needed to count repetitions and measure time or distance. Although these tests are more practical than maximal exercise testing, they have limitations. For example, during the 6MWT, the clinician can miss a lap, resulting in an underestimated walking distance. During the STS and step tests, repetitions or steps may be missed, and timing during the TUG can be affected by observer delay when starting or stopping the stopwatch.

Additionally, testing functional capacity in clinical settings has its limitations. In-clinic tests entail a serious risk that these are not adequately representative of how a person is functioning in daily life. In addition, episodic visits offer only a snapshot perspective, and are not suited to detect the day-to-day and within-day variations that are typical of daily life functioning. Such fluctuations often occur, for example related to medication intake or changes in environmental factors (e.g., temperature and air humidity) [8, 11, 19, 20].

Testing functional capacity at home may help overcome the above-mentioned limitations. Wearable technology can play an important role in achieving such remote, home-based testing [21–25]. By allowing frequent and repeated measurements in a person’s natural environment, these devices provide a more representative picture of daily life functioning and allow functional capacity to be tracked over longer periods of time [26, 27]. Because measurements are automatically recorded by the device, observer bias is minimized, increasing the accuracy and consistency of the main outcomes. Depending on the system used, data can either be transmitted securely to a cloud-based platform or server, or stored on the user’s device for later transmission. This enables remote monitoring by caregivers or researchers and facilitates longitudinal analysis. Such approaches may lower the barrier for patients to monitor their performance on a regular basis, while also reducing the barrier for caregivers and researchers. In addition, measuring functional capacity at home offers new opportunities to evaluate treatment effectiveness in daily life. In recent years, several functional capacity tests, supported by technology, have been developed for at-home use [27–30].

Here, we systematically review home-based functional capacity tests that have been described in the literature, focusing specifically on the 6MWT, TUG, STS, and step test, and we report on their clinimetric properties. With this overview, we provide further insight into how functional capacity can be assessed at home in different populations. We report on what is already known, what the knowledge gaps are and offer recommendations for future studies and practical applications.

## Methods

### Search Strategy

An electronic search in PubMed and CINAHL, was performed on January 15th, 2026 to find relevant articles. We used PubMed and CINAHL because our review focused on clinical validation studies, which are primarily indexed in biomedical and allied-health databases. While digital tests may also appear in technical or engineering databases, these typically focus on development or technical aspects rather than clinical validation. Since the aim of this review was to examine the clinical aspect specifically, PubMed and CINAHL were considered the most appropriate sources. Studies assessing the clinimetric properties (i.e. validity, reliability, and responsiveness) of remotely performed functional capacity tests were included in this review. Additionally, the tests needed to be performed in a population with a chronic condition. The search string used can be found in Table 1.

**Table 1:** Search string

Search domains	Search string PubMed	Search string CINAHL
Population	(“Cardiovascular Diseases“[Mesh] OR “Lung Diseases“[Mesh] OR “Neoplasms“[Mesh] OR “Nervous System Diseases“[Mesh] OR “Parkinsonian Disorders“[Mesh] OR “Surgical Procedures, Operative“[Mesh] OR cardiovascular disease*[tiab] OR COPD[tiab] OR elective surger*[tiab] OR heart disease*[tiab] OR lung disease*[tiab] OR neoplasm*[tiab] OR neurological disease*[tiab] OR oncolog*[tiab] OR Parkinson*[tiab] OR surgical procedure*[tiab]) AND	(MH “Cardiovascular Diseases+” OR MH “Lung Diseases+” OR MH “Neoplasms+” OR MH “Nervous System Diseases+” OR MH “Parkinson Disease+” OR MH “Surgical Procedures, Operative+” OR TI cardiovascular disease* OR AB cardiovascular disease* OR TI COPD OR AB COPD OR TI elective surger* OR AB elective surger* OR TI heart disease* OR AB heart disease* OR TI lung disease* OR AB lung disease* OR TI neoplasm* OR AB neoplasm* OR TI neurological disease* OR AB neurological disease* OR TI oncolog* OR AB oncolog* OR TI Parkinson* OR AB Parkinson* OR TI surgical procedure* OR AB surgical procedure*) AND
Home-based/remote	(“Mobile Applications“[Mesh] OR “Self Administration“[Mesh] OR mobile[tiab] OR remote[tiab] OR telemed*[tiab] OR technolog*[tiab] OR wearable*[tiab] OR home[tiab]) AND	(MH “Mobile Applications+” OR MH “Self Administration+” OR TI mobile OR AB mobile OR TI remote OR AB remote OR TI telemed* OR AB telemed* OR TI technolog* OR AB technolog* OR TI wearable* OR AB wearable* OR TI home OR AB home) AND
Functional capacity test	(“Exercise Test“[Mesh] OR exercise test*[tiab] OR capacity test*[tiab] OR physical test*[tiab] OR walking test*[tiab] OR walk test*[tiab] OR step test*[tiab] OR sit to stand[tiab] OR timed up and go[tiab] OR remote patient monitoring[tiab]) AND	(MH “Exercise Test+” OR TI exercise test* OR AB exercise test* OR TI capacity test* OR AB capacity test* OR TI physical test* OR AB physical test* OR TI walking test* OR AB walking test* OR TI walk test* OR AB walk test* OR TI step test* OR AB step test* OR TI sit to stand OR AB sit to stand OR TI timed up and go OR AB timed up and go OR TI remote patient monitoring OR AB remote patient monitoring) AND
Clinical trials	((clinical[tiab] AND trial[tiab]) OR clinical trials as topic[Mesh] OR clinical trial[pt] OR random*[tiab] OR random allocation[Mesh] OR cohort[tiab] OR validation[tiab] OR (intervention[tiab] AND stud*[tiab]))	((TI clinical AND AB trial) OR (TI clinical AND TI trial) OR (AB clinical AND AB trial) OR MH “Clinical Trials+” OR PT Clinical Trial OR TI random* OR AB random* OR MH “Random Assignment+” OR TI cohort OR AB cohort OR TI validation OR AB validation OR (TI intervention AND AB stud*) OR (AB intervention AND AB stud*) OR (TI intervention AND TI stud*))

### Inclusion and Exclusion Criteria

Studies aiming to assess one or more clinimetric properties (i.e. validity, reliability, or responsiveness) of their chosen method were eligible to be included in this review. Studies on people with any type of cancer, chronic cardiovascular, pulmonary, or neurological disorder or people undergoing elective surgery were eligible for inclusion in this review. Additionally, studies should have used an unsupervised or remotely supervised functional capacity test. Unsupervised tests were performed by the participants themselves, whereas remotely supervised tests were performed by the participants with guidance of a clinician via videoconferencing or telephone. Only original, full-text articles published in English were included in the review. Exclusion criteria were: review articles, design studies, case studies or studies using only questionnaires. Moreover, articles that used a functional capacity test to evaluate an intervention rather than to evaluate the measurement properties were excluded.

### Study Selection

**Fig. 1:**
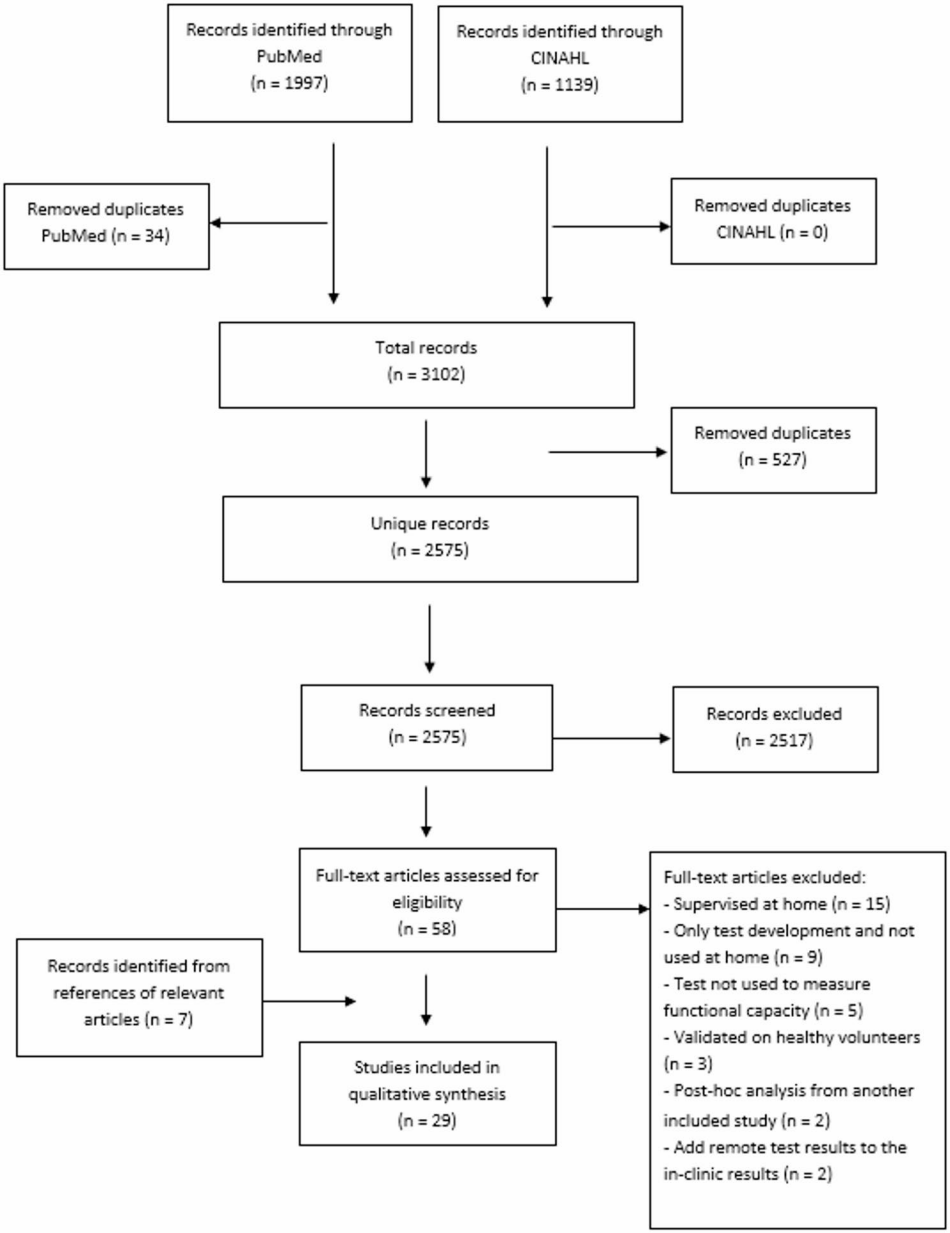
Study selection

We first screened the titles and abstract on the relevant subject. After that, a full text screening was performed using the same inclusion and exclusion criteria. References of the relevant articles were screened on title and included if they met the inclusion and exclusion criteria (Fig. 1). Screening and selection was performed by two independent raters (DdG and SMMT). Any disagreement was resolved in a consensus meeting. The process of including studies was executed using the web application of Rayyan [31]. The review was carried out according to the PRISMA guidelines [32].

### Data Extraction

Relevant information including study design, study population, methods of the study and type of functional capacity test was extracted and registered. A risk of bias assessment on the included studies using the Consensus-based Standards for the selection of health Measurement Instruments (COSMIN) was conducted [33]. The COSMIN checklist is a tool to assess the quality of studies on measurement properties [34].

To complete the COSMIN checklist, two independent raters (DdG and SMMT) graded the methodological quality. The risk of bias assessment consists of multiple sections that refer to several aspects of measurement properties (e.g., structural validity and content validity). Only those sections that applied to the particular study were completed [35]. The items were assessed using four scores outlined by COSMIN: very good, adequate, doubtful or inadequate. The overall quality assessment of a study was determined using ‘the worst score counts’ principle, e.g., if one item was scored as “inadequate”, the overall score of the development and risk of bias assessment would be “inadequate” [34]. If the decisions between the raters differed, they discussed their ratings and decided on the final score together.

Information about reliability, validity and responsiveness was extracted and registered, when reported in the article. In this context, validity was defined as how well the scores on the unsupervised functional capacity test matched those of the supervised functional capacity test. Test-retest reliability was defined as how consistent repeated scores were when measured within the same person on different occasions. Responsiveness was defined as the degree to which the functional capacity test can detect clinically important changes over time. Validity, reliability and responsiveness were rated as positive when the intraclass correlation coefficient or weighted Kappa was ≥ 0.70 in a sample size of at least 50 patients [36]. Studies were rated “+” when they met the criterion, “-” if they did not meet the criterion, “?” when the result was doubtful and “0” when there was no information available [36, 37]. When other statistical measures were reported (e.g., Pearson or Spearman correlations, coefficient of variation, mean differences, or variance measures), ratings were based on predefined thresholds for acceptable measurement performance. Correlation coefficients were rated as positive when they exceeded 0.70, while coefficients of variation < 5% were considered indicative of good reliability. For agreement analyses, a positive rating was assigned when measurement error was small relative to the mean test value and reported clinically meaningful differences. The authors’ conclusions regarding measurement performance were also taken into account. This approach allowed inclusion of heterogeneous outcome metrics while maintaining consistent qualitative ratings across studies. In this review, information on feasibility and usability was described when reported in the included studies. A functional capacity test is feasible when the tool is suitable for the target population and can reasonably be performed at home. Available information about usability is described, where a score of 80 and higher on the system usability scale (SUS) is considered above average and indicates that the approach is usable [38].

## Results

The search yielded 1997 articles from PubMed and 1139 articles from CINAHL. After removal of duplicates and screening on title and abstract, 58 articles were selected and full texts were assessed for eligibility (Fig. 1). Seven additional articles from reference lists of relevant articles were added. After screening of the full text articles, we included 29 articles. Table 2 shows the characteristics of the included studies. In Table 3, we present the ratings of reliability, validity and responsiveness together with additional information on feasibility, usability or other relevant outcomes. The results are categorized based on the functional capacity tests that were included: the 6MWT, TUG, 30sSTS and step test.

**Table 2:** Characteristics of the included studies

Study	Study design	Aim	Diagnosis (*n*)	Functional capacity test	Supervised or unsupervised	Test materials
Brooks et al. [10]	Observational	Validation	CHF and PH (19 for home validation)	6MWT	Unsupervised	Smartphone application with video instructions on how to perform the 6MWT at home.
Burch et al. [47]	RCT	Validation	CHF (197)	6MWT	Unsupervised	Wearable cardioverter defibrillator (WCD)-guided 6MWT gave verbal and video instructions to start and stop the test. Patients could walk in a circle, oval, square, rectangle, or straight line.
Cox et al. [60]	Crossover	Feasibility	CF (10)	3-min step test	Remotely supervised	Videoconferencing to supervise the test. 15 cm high step, 30steps/min for 3 min. paced using a metronome.
Cox et al. [61]	Prospective cohort	Validity, safety, acceptability	Chronic pulmonary disease (38)	Incremental step test	Remotely supervised	Videoconferencing to supervise the test. 20 cm high step, stepping rate externally paced using digital audio-recording. Start-pace is 10steps/minute, one step added every 30 s. Test ends if participant is unable to keep pace with the signal for 15 s.
Douma et al. [48]	Cross-sectional	Feasibility, validity, reliability	Cancer (72)	6MWT	Unsupervised	Smartphone application using GPS to measure distance walked. The test was performed outdoors.
Douma et al. [52]	Observational	Effectiveness	Cancer (136)	6MWT	Unsupervised	Smartphone application using GPS to measure distance walked. The test was performed outdoors.
Du et al. [7]	Cross-sectional	Validity	CHD (29)	6MWT	Unsupervised	5-m long rope was laid out flat on a hard surface. A lap counter was used to measure distance walked in 6 min.
Du et al. [51]	RCT	Effectiveness	CHF (132)	6MWT	Unsupervised	5-m long rope was laid out flat on a hard surface. A lap counter was used to measure distance walked in 6 min.
Grobe-Einsler et al. [55]	Prospective cohort	Feasibility, usability	PD (25)	TUG	Unsupervised	Videorecording of the TUG that is uploaded to the researchers via smartphone app. Each session, 3 TUGs were performed, for 12 weeks. Mean of these trials was used as representation of that week.
Hameed et al. [57]	Prospective cohort	Effectiveness	COVID-19 (106)	30sSTS 2-min step test	Remotely supervised	Videoconferencing used to supervise the test. 30sSTS test was performed by counting the number of times patients could stand up from a chair with their arms folded across their chest. 2-min step test was performed by letting the patients stand up and step in place.
Hwang et al. [39]	Cross-sectional	Validity	CHF (17)	6MWT TUG	Remotely supervised	Videoconferencing was used to supervise tests at the hospital. 6MWT was performed in a 30-meter corridor while the examiner counted the laps. The TUG was performed on a 45-cm high chair, a stopwatch was used to monitor the time.
Jehn et al. [49]	RCT	Effectiveness	COPD (62)	6MWT	Unsupervised	Accelerometry on a suitable flat walking path. Distance was measured with a distance wheel to assure accuracy of the walking trajectory.
Jehn et al. [40]	RCT	Feasibility	CHF (155)	6MWT	Unsupervised	Accelerometry on a suitable flat walking path. Distance was measured with a distance wheel to assure accuracy of the walking trajectory.
Juen et al. [41]	Cross-sectional	Validation	Pulmonary diseases (28)	6WMT	Unsupervised	Smartphone worn at L3 level running the smartphone application using accelerometer data.
Landers and Ellis [56]	Prospective cohort	Effectiveness	PD (28)	30sSTS TUG	Unsupervised	Commercially available smartphone app with video instructions and built-in timer.
Lopane et al. [69]	Pilot observational	Effectiveness	PD (20)	TUG	Unsupervised	Smartphone functioning as an IMU located on L5. Vocal, textual and video guidance is provided on how to wear the smartphone on the lower back.
Mak et al. [42]	Observational	Validity	Cardiovascular disease (110)	6MWT	Unsupervised	Smartphone application using GPS data. The test was performed in an open space at home.
Mavronasou et al. [58]	Prospective	Validity	COVID-19 (25)	1mSTS Step test	Remotely supervised	Videoconferencing to supervise the test at home. At home, a caregiver was present for safety reasons. They counted the completed STS cycles to avoid miscounting. 17 cm high step for the step test was used. 5 stages, each 2 min with increasing cadence of 5 steps/minute set by a metronome. Start cadence was 15 steps/minute.
Motolese et al. [70]	Observational	Feasibility	PD (54)	TUG	Unsupervised	Smartphone application with integrated video instructions for the TUG.
Prescher et al. [50]	RCT	Intervention	CHF (155)	6MWT	Unsupervised	Telemonitoring using an accelerometer on a suitable walking path identified by a nurse. Distance was measured with a distance wheel to assure accuracy.
Salvi et al. [43]	Observational	Validity	PH (30)	6MWT	Unsupervised	Smartphone application that can be used indoors and outdoors. GPS was used for outdoor measurements. For indoor measurements the inertial sensors were used to measure the number of U-turns while walking on a straight walkway.
Saporito et al. [53]	Cross-sectional	Validity	THA (15)	TUG	Unsupervised	IMU worn in front of the chest was used to determine TUG from data measured during free-living activities.
Scherrenberg et al. [44]	Observational	Validity	Cardiovascular disease (102)	6MWT	Unsupervised	Smartphone app using GPS and Google Fit software to determine distance walked in 6 min.
Sokas et al. [71]	Observational	Feasibility	Cardiovascular disease (28)	6MWT	Unsupervised	Wrist-worn device (Fitbit) using calculated step data.
Stienen et al. [45]	Cross-sectional	Validity	Spine-related disability (406)	6MWT	Unsupervised	Smartphone application was used in an optimal environment close to the home without obstacles. The application used GPS and was specifically designed for this population.
Trymbulak et al. [72]	Pilot observational	Feasibility	Atrial fibrillation (40)	6MWT	Unsupervised	Fitbit device and application on a smartphone with automated messages that communicated the start and stop times of the walk test. The test was performed on a flat surface.
Wevers et al. [46]	Cross-sectional	Validity	Stroke (27)	6MWT	Supervised for instructions, unsupervised for data collection	Handheld GPS and measuring wheel that measured the distance of the 6MWT performed outdoors.
Wickerson et al. [62]	Prospective	Validity	Advanced lung diseases (24)	6MWT	Remotely supervised	Videoconferencing to supervise the test. Same standard phrases according to 6MWT guidelines were used. Caregiver present to mark the end position
Zampieri et al. [54]	Pilot cross-sectional	Feasibility	PD (6) and healthy controls (8)	TUG	Unsupervised	Patients wore a portable data-logger on a waist belt with five inertial sensors attached to their body. The distance that needs to be walked was 7 m and should be on a flat surface.

*1mSTS* 1 min Sit-To-Stand test, *6MWT* 6 min Walk Test, *30sSTS* 30 s Sit-To-Stand test, *CF* Cystic Fibrosis, *CHD* Coronary Heart Disease, *CHF* Chronic Heart Failure, *COPD* Chronic Obstructive Pulmonary Disease, *GPS* Global Positioning System, *IMU* Inertial Measurement Unit, *PD* Parkinson’s Disease, *PH* Pulmonary Hypertension, *RCT* Randomized Controlled Trial, *THA* Total Hip Arthroplasty, *TUG* Timed Up and Go, *WCD* Wearable Cardioverter Defibrillator

**Table 3:** Clinimetric properties of functional capacity tests

Study	Functional capacity test	Validity	Reliability	Responsiveness	Quality of development	Risk of bias	Remarks
Brooks et al. [10]	6MWT	+	+	0	Very good	Very good	Application was easy to use independently.
Burch et al. [47]	6MWT	?	+	0	Very good	Very good	Not more fatigue after 6MWT at home than in-clinic.
Cox et al. [60]	3-min step test	0	0	0	Doubtful	Very good	System usability scale: 85.6 (95% CI 79.8–91.5). Most participants had no preference for in-person or remote testing.
Cox et al. [61]	Incremental step test	+	0	0	Very good	Very good	No difference in ceasing the test by the clinician. Preference for in-clinic testing was 42%, 18% preferred remote testing, 40% had no preference for test location
Douma et al. [48]	6MWT	-	+	0	Very good	Doubtful	Mean score SUS was 69.
Douma et al. [52]	6MWT	0	0	0	Adequate	Very good	Median usability score was 80 (59–90) out of 100.
Du et al. [7]	6MWT	+	+	0	Doubtful	Very good	Shortest distance walked in first attempt at home. Reported to be an enjoyable activity. Useful tool for motivation and goal setting.
Du et al. [51]	6MWT	0	0	?	Very good	Very good	Self-monitoring group improved in self-care behavior and physical activity level.
Grobe-Einsler et al. [55]	TUG	0	Within session: + Within weeks: ?	0	Adequate	Adequate	Mean SUS was 75.5%
Hameed et al. [57]	30sSTS and 2-min step test	0	0	30sSTS : + 2MST : ?	Inadequate	Very good	
Hwang et al. [39]	6MWT and TUG	6MWT: + TUG: +	6MWT: + TUG: +	0	Very good	Very good	System usability score was 85 (SD: 15) out of 100. Some technical issues were reported including internet dropout, video freezing and delays in transmission.
Jehn et al. [49]	6MWT	0	0	+	Inadequate	Inadequate	
Jehn et al. [40]	6MWT	+	+	0	Very good	Doubtful	59% completed 6 months of activity monitoring and 84% completed a minimum of 12 months
Juen et al. [41]	6WMT	+	0	0	Inadequate	Doubtful	
Landers and Ellis [56]	30sSTS and TUG	0	0	?	Doubtful	Doubtful	12/28 participants used the app more than 150 min per week. The other 16 participants used the app 120–150 min (n : 3), 90–120 (n : 4), and < 90 min (n : 9). Most participants found the app added value.
Lopane et al. [69]	TUG	0	0	0	Doubtful	Doubtful	95% of participants were satisfied with self-administration of the monitoring protocol.
Mak et al. [42]	6MWT	+	+	0	Very good	Inadequate	Only as iOS application available.
Mavronasou et al. [58]	1mSTS Step test	1mSTS: + Step test: +	0	0	Very good	Very good	No discrepancies observed between physiotherapist and third person for total number of repetitions during the 1mSTS.
Motolese et al. [70]	TUG	0	0	0	Inadequate	Doubtful	Construct validity: total TUG time was correlated with disease duration, UPDRS-III total score and Hoehn & Yahr score. 83.3% of participants used the app at least once; 53.7% of participants used the app once every week and 29.6% used the app twice every week.Technical issues or digital illiteracy (38.7%), demotivation (24%) and health-related issues (7.4%) were reasons for non-compliance.
Prescher et al. [50]	6MWT	0	0	+	Very good	Very good	
Salvi et al. [43]	6MWT	+	+	0	Very good	Doubtful	All participants performed the 6MWT at least once and 48% performed the 6MWT at least once every month. Acceptance and usability were rated positive.
Saporito et al. [53]	TUG	+	+	+	Adequate	Doubtful	
Scherrenberg et al. [44]	6MWT	+	+	0	Very good	Very good	Useful tool that contributes to the motivation of the participant.
Sokas et al. [71]	6MWT	0	0	0	Very good	Adequate	After 1 month, 82% of the CVD patients performed at least one 6MWT.
Stienen et al. [45]	6MWT	+	0	0	Inadequate	Inadequate	Free app available for Android and iOS.
Trymbulak et al. [72]	6MWT	0	0	0	Inadequate	Inadequate	After one month, 63% of participants completed the 6MWT, after 6 months this was 28%.
Wevers et al. [46]	6MWT	+	+	0	Very good	Adequate	
Wickerson et al. [62]	6MWT	-	0	0	Adequate	Inadequate	No differences in vital signs observed between in-clinic and remote 6MWT.
Zampieri et al. [54]	TUG	-	0	0	Adequate	Adequate	PD participants walked significantly slower at home.

*2MST* 2-min step test, *6MWT* 6 min Walk Test, *30sSTS* 30 s Sit-To-Stand test, *CF* Cystic Fibrosis, *CHD* Coronary Heart Disease, *CHF* Chronic Heart Failure, *COPD* Chronic Obstructive Pulmonary Disease, GPS Global Positioning System, *IMU* Inertial Measurement Unit, *PD* Parkinson’s Disease, *PH* Pulmonary Hypertension, *RCT* Randomized Controlled Trial, *THA* Total Hip Arthroplasty, *TUG* Timed Up and Go, *WCD* Wearable Cardioverter Defibrillator

+ : intra class correlation coefficient (ICC) or weighted Kappa was ≥ 0.70, - : ICC or weighted Kappa was < 0.70, ? : doubtful result based on ICC or weighted Kappa, 0 : no information available about this metric

Correlations > 0.70 and coefficients of variation < 5% were rated positive. Agreement was judged based on measurement error relative to test values and clinically meaningful differences, considering authors’ conclusions

### 6MWT

The home-based 6MWT was the most widely used test across studies (*n* = 19). To estimate the covered distance, both sophisticated technologies and more traditional tools are used. Sophisticated technologies rely on global positioning system (GPS), accelerometer data and/or inertial sensors to estimate walking distance, whereas more traditional tools include a rope or a hand-mounted lap counter. The 6MWT has been studied in different populations, including people with cardiovascular and pulmonary diseases (Table 2). Only six studies (32%) scored ‘very good’ on both the quality of development and risk of bias items of the COSMIN checklist, whereas eight studies scored ‘very good’ on one of these items (Table 3).

In total, 13 studies (65%) investigated the validity of their remote 6MWT. Ten of these (77%) reported that their method was a valid approach [7, 10, 39–46]. Smartphone applications were found to be valid in six studies [10, 41–45], while videoconferencing [39], accelerometry [40], GPS [46], and a rope with lap counter [7] were each found to be valid in one study. One study reported a doubtful validity using the wearable cardioverter defibrillator (WCD)-guided app [47]. In the latter study, a statistically significant difference of 15 steps was observed between the first in-clinic 6MWT and the first home-based 6MWT (558 vs. 543 steps, *p* = .001). However, this difference represents less than 3% of the average step count per 6MWT and was considered unlikely to be clinically meaningful by the authors. No differences were found between the app-based and traditional 6MWTs performed in-clinic. A similar pattern emerged when assessing the reliability. Nine studies (45%) investigated reliability, with smartphone applications shown to be reliable in four studies [10, 42, 43, 48], while videoconferencing [39], the WCD-guided app [47], accelerometry [40], GPS [46], and the rope with the lap counter [7] were each found to be reliable in one study (Table 3). Notably, the studies that presented a reliable result also showed proof of validity.

Responsiveness was barely examined. Two studies reported positive outcomes related to responsiveness, both using accelerometry [49, 50]. One study reported on responsiveness, but their results showed that no changes over time were measured using a rope and the distance wheel. After a six-month intervention, only a slight increase in the 6MWT distance was found, which was not significant [h^51^] (Table 3).

Two of the included studies reported on the usability of their approach by using the SUS which measures the usability of electronic systems, covering videoconferencing (SUS score 85 out of 100) [39] and smartphone-based approach (SUS score 80 out of 100) (Table 3) [52]. Both found their approach to be usable.

### TUG

Seven studies were identified that used an unsupervised or remotely supervised home-based TUG assessment in people with Parkinson’s disease (*n* = 5), people with cardiovascular diseases (*n* = 1) and people that underwent total hip arthroplasty (*n* = 1). These studies employed different methods, including smartphone applications and inertial measurement units (Table 2).

In total, three studies investigated the validity of the home-based TUG. Two of the studies concluded that their videoconferencing [39] and IMU [53] approach was valid. The validity of one study when using five inertial sensors attached to the body was reported as insufficient, as the participants walked significantly slower at home compared to their in-clinic walking speed [54]. Three studies investigated the reliability. Videoconferencing [39] and IMU-based [53] approaches were found to be reliable, whereas one study was rated as doubtful due to good within-session but inconsistent within-week measurements [55]. Responsiveness was investigated by two studies, of which one study reported a sufficient responsiveness with their IMU-based approach [53]. The second study, which used a smartphone application, found a statistically significant improvement in speed after eight weeks. However, the change from 11.2 to 8.5 s was below the clinically meaningful difference of 4.85 s [56], resulting in a doubtful result for responsiveness. The studies by Hwang et al. and Grobe-Einsler et al. also examined usability and found that their smartphone-based [39] and videoconferencing [55] approaches were usable for all users.

### 30sSTS

Three studies investigated the sit to stand test in a home-based setting using a commercial available smartphone application and videoconferencing to supervise the test [56–58] (Table 2). Only Mavronasou et al. evaluated the validity of their videoconferencing approach in participants with COVID-19 and found it to be valid [58]. None of the studies investigated reliability. The videoconferencing approach of Hameed et al. demonstrated sufficient responsiveness in their COVID-19 population [57]. The smartphone application used by Landers et al. [56] showed a statistically significant change over eight weeks. However, the increase from 11.6 to 14.3 steps resulted in a doubtful score as it was just slightly below the clinically meaningful difference of 3 steps [59].

### Step Test

Four articles studied a home-based step test using videoconferencing [57, 58, 60, 61] (Table 2). Incremental step tests were investigated in two studies and found to be valid [58, 61]. None of the studies assessed reliability. One study evaluated the 2-minute step test, for which responsiveness was classified as doubtful. The results showed that 65% of the participants improved their physical functioning after two weeks of training, but that was insufficient to be conclusive about responsiveness [57]. The study investigating the 3-minute step test reported that their videoconferencing approach was usable, with a SUS score of 85.6 out of 100 [60] (Table 3).

## Discussion

In this study, we systematically reviewed current knowledge with respect to the clinimetric properties of testing functional capacity at home. In recent years, there has been a marked increase in studies assessing functional capacity at home. These tests were either completely unsupervised or were assisted remotely. This trend is most likely driven by advances in wearable technology, with a further impetus by the COVID-19 pandemic which precluded in-clinic assessments. The clinimetric properties of the 6MWT were studied most widely in the 29 included articles in this review, showing acceptable results with respect to validity and reliability. Less information was available for the clinimetric properties of the TUG, 30sSTS and step test. Yet, it is difficult to draw firm conclusions, as the methodological quality with which most studies had been carried out was limited.

The 6MWT was investigated most widely, with 19 studies using various approaches. Notably, the eight studies with the best methodological quality focused on this test. The 6MWT using smartphone applications (by either deploying the embedded accelerometer or the GPS) showed acceptable results in six studies. Despite the fact that the method of these approaches was similar, i.e. using a smartphone app, each study deployed a different app. This diversity is actually a promising indicator that the 6MWT can be performed using different smartphone applications, which argues in favor of its generalizability. Even though most of the smartphone apps that we reviewed showed acceptable results with respect to validity and reliability, this might not apply to other existing or forthcoming apps that have thus far not been tested. It is therefore essential to establish the validity and reliability of any new app before deploying it in research and clinical practice, despite these promising results.

The study by Burch et al. [47] used a smartphone application to guide the 6MWT, but their approach was found not to be valid, as a significant difference existed between the first home-based 6MWT and the in-clinic 6MWT. The same pattern was observed in the study by Wickerson et al. [62]. However, the in-clinic and home-based test protocol differed from each other, potentially explaining this difference. The in-clinic test was performed according to the American Thoracic Society guidelines for the 6MWT, with a walking course of 30 m straight [63], which is difficult to achieve at home. For the at-home measurements in the study by Burch et al., participants were instructed to always use the same location to perform the test [47]. However, participants could determine the course shape and course length themselves; they were allowed to walk in an oval, square, rectangle or in a straight line but the authors reported that they did not have insight in the chosen courses. Similarly, in the study by Wickerson et al. [62], participants were instructed to walk on the same 10 m straight walkway inside their homes. This may explain the significant difference between the in-clinic and home-based 6MWT, since it is known that changing course shape and course length has a significant effect on the distance walked during the 6MWT. The distance walked during the 6MWT significantly decreases when the course is shorter, leading to an underestimation of the walking distance. Changing course shape and decreasing the amount of turns and the angle of the turn increases the ability to keep walking speed stable throughout the test. This, in turn, increases the walking distance and may lead to an overestimation of the walking distance [64–66]. In addition to course layout, practical aspects of the home environment may further influence performance. Smaller indoor tracks with narrow hallways, doorways and walking surfaces such as carpets may affect maneuverability. This can increase surface friction, particularly for participants using oxygen equipment or walking aids, potentially resulting in shorter walking distances during the 6MWT [62]. Some studies have overcome this issue by testing outdoors over a straight 30 m course, but a suitable path without obstructions is needed and the weather may influence the results [67, 68]. Therefore, careful consideration must be given to course layout and environmental factors when conducting the 6MWT at home.

Little evidence on responsiveness was available for the remote 6MWT, which is essential when the intended use of an assessment tool is to evaluate effects of an intervention. Two studies reported that their remote 6MWT approach was able to detect changes over time.

Specifically, one study investigated responsiveness in persons with chronic heart failure who were tested before and after a 6-month intervention where participants had to perform a home-based unsupervised 6MWT on a daily basis, but their approach did not find any differences [51]. Performance was slightly increased after three months, but this improvement was not sustained after the entire six months of training. Moreover, the distance walked during the 6MWT (as measured by the clinician) did not change after the intervention period. This might indicate that the intervention itself was not able to increase functional capacity. The authors argue that the lack of effects might be explained by the participants’ characteristics. On average, participants walked about 30 m more during the 6MWT at baseline compared to other studies that were performed in the same population. This higher baseline 6MWT distance could have led to a ceiling effect, which may explain why the 6MWT distance did not increase significantly over the study period. Additionally, participation in this study was voluntary, and it was thus likely that participants were more interested in self-monitoring [51].

There is little evidence to draw conclusions on the validity and reliability of the TUG at home. For the 30sSTS and step test there is even less evidence available. However, compared to the 6MWT, the TUG, 30sSTS and step test are easy to perform as they require little space. Additionally, these tests take a shorter time to complete which may improve compliance with home-based testing.

It is notable that not even one third of the included studies in this review scored ‘very good’ on one of the COSMIN checklist items. This might indicate that the methods to assess the clinimetric properties of a certain approach might not be optimal for digital tools. However, there is no validated checklist yet to assess the methodological quality of different digital tools to obtain functional capacity at home. We decided to use the COSMIN checklist, since that checklist comes closest. However, this checklist was originally created to assess the methodological quality of patient reported outcome measures, but the techniques that were used to measure functional capacity at home were objective measures and not necessarily patient reported outcomes. The items on the COSMIN checklist did not always apply to the used materials which made scoring the items sometimes difficult, which is a limitation of this review.

Overall, the findings of this review show that several digitally supported functional tests can be performed in the home environment. However, practical implementation requires attention to both technical and human factors. Many approaches rely on stable internet access and external platforms for data processing, storage, or review, which introduces dependence on remote servers and digital infrastructure. In addition, effective use of these tools may depend on a certain level of digital skills and confidence with technology, which cannot be assumed in all chronic disease populations. Digital tools should therefore not automatically be considered superior to simpler, low-tech alternatives, which may in some contexts be more robust and easier to implement.

Participant experience also affects test outcomes and should be considered alongside measurement objectives. In-clinic testing may yield slightly better scores due to both verbal and non-verbal encouragement [63]. In contrast, remote supervision is typically limited to verbal cues and may lead to underestimation of exercise capacity. Expectations or anxiety related to performing the test at home without in-person supervision may further influence pacing behavior and perceived exertion [62]. Participant preferences for testing location may affect engagement and experience. Two of the included studies investigated these preferences. Cox et al. reported that 42% of participants preferred in-clinic testing, 18% preferred remote testing, and 40% expressed no preference in a population with chronic respiratory disease [61]. Similarly, Scherrenberg et al. found that 27% favored in-clinic testing, 29% preferred remote testing, and 43% had no clear preference in a population with cardiovascular disease [44]. Taken together, these finding show that preferences vary across individuals and populations, and that no single setting is consistently favored.

Beyond participant experience and preferences, the terminology used in the literature also warrants consideration. Assessments labelled as unsupervised are not always performed without support, and may include remote observation or guidance via videoconferencing. Digitally supported testing should therefore be seen as a spectrum, ranging from fully automated applications to real-time supervision at a distance using digital tools.

Finally, the choice of testing method may depend on the intended clinical purpose. In-clinic testing may be preferable when the goal is to obtain the most accurate estimate of maximal exercise capacity. Home-based testing, on the other hand, may be more suitable for longitudinal monitoring, such as evaluating natural progression or the effects of an intervention over time. In the home-based context, test conditions can be standardized across sessions while reducing participant burden and potentially improving adherence. Taken together, these findings highlight that successful implementation of home-based functional capacity testing depends not only on measurement properties, but also on participant comfort, motivation, supervision, and the intended clinical purpose of the assessment.

A major strength of this review is the broad scope of investigated functional capacity tests using various approaches in a wide range of chronic conditions. We did not limit ourselves to specific tests or to certain populations. With that strategy, we were able to provide valuable insights about the clinimetric properties of functional capacity tests in chronic conditions. The 6MWT showed promising results when using smartphone applications in people with chronic diseases, although more research with good methodological quality is needed to confirm these findings.

## Conclusion

Testing functional capacity at home using various approaches is advancing rapidly, driven by technological innovations and an increasing recognition of monitoring individuals within their own environment. However, the clinimetric properties should be evaluated and proven sufficient before these approaches can be used in research and clinical practice. It must be ensured that the research methodology is of good quality.

## Supplementary Information

Below is the link to the electronic supplementary material.

Supplementary Material 1.

## Data Availability

The datasets used and analyzed during the current study are available from the corresponding author on reasonable request.

## Abbreviations

6MWT: 6 min walk test
30sSTS: 30 s sit-to-stand test
CF: Cystic fibrosis
CHD: Coronary heart disease
CHF: Chronic heart failure
COPD: Chronic obstructive pulmonary disease
COSMIN: Consensus-based standards for the selection of health measurement instruments
GPS: Global positioning system
IMU: Inertial measurement unit
PD: Parkinson’s disease
PH: Pulmonary hypertension
RCT: Randomized controlled trial
STS: Sit to stand test
THA: Total hip arthroplasty
TUG: Timed up and go
WCD: Wearable cardioverter defibrillator
