# Supplementary material for: Unsupervised Functional Capacity Testing at Home: A Systematic Review
Source: Sports Med Open. 2026 May 27;12:57. doi: 10.1186/s40798-026-01029-6 (PMC13216383; doi:10.1186/s40798-026-01029-6)
Supplement: Supplementary file 1 — Supplementary Material 1. [file 40798_2026_1029_MOESM1_ESM.pdf]

Supplementary Table 1: Numerical summary of clinimetric properties of functional capacity tests

Study	Functional capacity test	Validity	Reliability	Responsiveness
Brooks et al. [10]	6MWT	ICC 0.89	Coefficient of variation = 4.6%	NA
Burch et al. [47]	6MWT	Clinician vs app: 306 vs 297 meters (t(176) = 0.64, P =.53)	Wilcoxon signed-rank test week to week not significant (after Holm method correction)	NA
Cox et al. [60]	3-min step test	NA	NA	NA
Cox et al. [61]	Incremental step test	ICC 0.93	NA	NA
Douma et al. [48]	6MWT	ICC 0.47	ICC 0.88	NA
Douma et al. [52]	6MWT	NA	NA	NA
Du et al. [7]	6MWT	Correlation coefficient r = 0.99	ICC 0.98	NA
Du et al. [51]	6MWT	NA	NA	After six months no significant difference $F(1,129) = 0.052$, $p = 0.820$
Grobe-Einsler et al. [55]	TUG	NA	Within session: variance 0.87 s^2 Within weeks: variance 12.55 s^2	NA
Hameed et al. [57]	30sSTS<br>2-min step test	NA	NA	30sSTS: 65-88% (therapy groups) and 17% (no therapy) reached clinically meaningful difference<br>2-min step test: 50-74% (therapy groups) and 50% (no therapy) reached clinically meaningful difference

Hwang et al. [39]	6MWT<br>TUG	6MWT: ICC 0.90<br>TUG: ICC 0.85	6MWT: ICC 0.99<br>TUG: ICC 0.95	NA
Jehn et al. [49]	6MWT			Mean Difference in 6MWT: +87.0 ± 65.7 meters, *p* = 0.006
Jehn et al. [40]	6MWT	Correlation coefficient r = 0.90	Correlation coefficient of r = 0.89	NA
Juen et al. [41]	6WMT	Method error = 3.8%	NA	NA
Landers and Ellis [56]	30sSTS<br>TUG	NA	NA	30sSTS: 11.6 to 14.3 < meaningful clinical difference<br>TUG 11.2 to 8.5 < meaningful clinical difference
Lopane et al. [69]	TUG	NA	NA	NA
Mak et al. [42]	6MWT	Mean error = 5.6%	Cronbachs alpha 0.99 (95% CI: 0.988–0.992)	NA
Mavronasou et al. [58]	1mSTS<br>Step test	1mSTS: ICC = 0.977<br>Step test: ICC = 0.871	NA	NA
Motolese et al. [70]	TUG	NA	NA	NA
Prescher et al. [50]	6MWT	NA	NA	AUC = 0.73 (95% CI 0.63–0.83)
Salvi et al. [43]	6MWT	Correlation coefficient r = 0.89	ICC 0.91	NA
Saporito et al. [53]	TUG	Correlation coefficient r = 0.7	ICC 0.94	After 12 weeks *p* < 0.001
Scherrenberg et al. [44]	6MWT	Mean difference 66 meters	NA	NA
Sokas et al. [71]	6MWT	NA	NA	NA
Stienen et al. [45]	6MWT	ICC 0.97	NA	NA
Trymbulak et al. [72]	6MWT	NA	NA	NA
Wevers et al. [46]	6MWT	ICC 0.98	Correlation coefficient	NA

			r = 0.98	
Wickerson et al. [62]	6MWT	Mean difference 56m > clinically important difference	NA	NA
Zampieri et al. [54]	TUG	$p < 0.05$	NA	NA
Abbreviations: 1mSTS = 1 Minute Sit-To-Stand test, 30sSTS = 30 Second Sit-To-Stand test, 6MWT = 6-Minute Walk Test, AUC = Area Under Curve, ICC = Intraclass Correlation Coefficient, NA = not applicable, TUG = Timed-Up-and-Go test.				
